# *In situ* electrochemical monitoring with an open circuit auxiliary electrode in microbial electrochemical cells treating sediments†

**DOI:** 10.1039/d5ra03133h

**Published:** 2025-06-25

**Authors:** Carlos Sánchez, Amitap Khandelwal, Piet N. L. Lens

**Affiliations:** a University of Galway University Road H91 TK33 Ireland piet.lens@universityofgalway.ie

## Abstract

A set of six low-cost 3D printed microbial electrochemical cells (MECs), each containing four electrodes and one ceramic membrane were constructed. The electrodes included a polarized working electrode (WE, polarized at +0.2 V *vs.* Ag/AgCl), a reference electrode (RE) and a counter electrode (CE) typical in three-electrode electrochemical cells and an additional fourth electrode *i.e.*, auxiliary electrode (AE). The AE was identical to the WE, but it was kept in open circuit. This study was conducted to evaluate the potential of AE as a measurement of the bulk potential and control for the effect of polarization of the WE. Two different salinity levels, *i.e.*, 12 and 50 mS cm ^−1^ were tested in triplicate along with propionate concentrations of 0.1 g L^−1^ and 1 g L^−1^ to study the oxidation of propionate by the microbial community present in the reactor. Continuous monitoring of the electrode potentials, cyclic voltammetry (CV) at different scan rates and electrochemical impedance spectroscopy (EIS) data were obtained throughout the experiment to compare the results from the AE and the WE. The open-circuit auxiliary electrode (AE) was useful to control the state of the electrolyte and to distinguish changes in the system caused by the continuous polarization. The use of the AE allowed to compare the changes in the EIS diffusion slope caused by the WE polarization. This study showed that AE in MECs helps to understand and predict the electrochemical reactions more precisely.

## Abstract 2

Deployment of open circuit auxiliary electrodes in microbial electrochemical cells enables diverse applications in energy, agriculture, and environmental systems.

## Graphical abstract

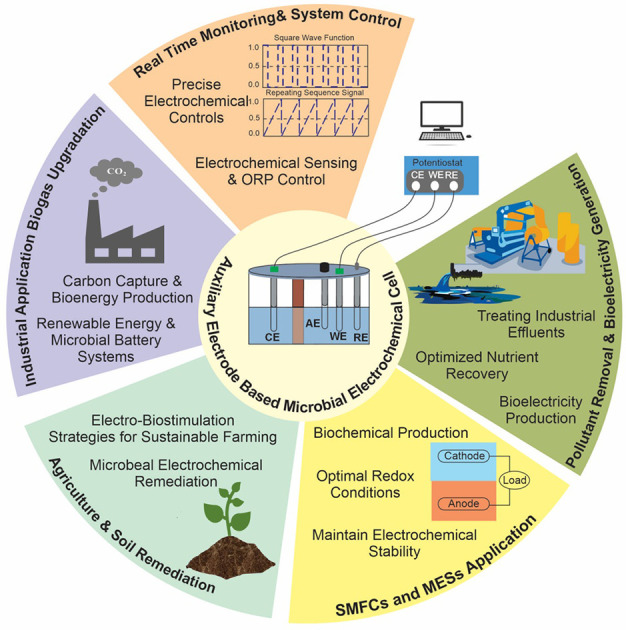

## 1 Introduction

Microbial electrochemical technologies (METs) are promising technologies that combine electrochemical processes with the electroactive catalytic capabilities of microorganisms.^1,2^ METs applications include electrical current production from waste oxidation in microbial fuel cells (MFCs)^3–5^ or to produce molecules from CO_2_ fixation in microbial electrosynthesis cells (MESs).^6^ However, METs are not yet mature^7^ and the experimental studies lack sufficient standardization.^8^ In METs electroactive microorganisms grow by profiting from the energy difference between two distinctive redox levels: the bulk electrolyte potential and the electrode potential.^9^ The electrical potential of bulk electrolyte with respect to a reference electrode is referred to as bulk electrolyte potential.^9,10^ MFCs consist of two simultaneous electrode–electrolyte reactions that mutually affect each other. As microbial activity can induce variability between replicates and experiments, real-world MET applications are difficult to implement and troubleshoot. An alternate approach is to control the electrode potential and reduce the variability caused by changes in electrode potential.^11,12^ A typical potential-controlled MET experiment involves the use of a potentiostat and a three-electrode cell which includes a working electrode (WE), a reference electrode (RE), and a counter electrode (CE).^13^ The use of this setup allows one to set a constant potential to the WE in relation to the RE and use the CE to close the circuit.

The variability of media composition is another important parameter due to its effect on the bulk redox potential values and the microbial activity.^14^ It was observed that dissolved organic carbon and ammonium concentration were strongly corelated to changes in microbial community structure. Thus, changes in the bulk potential, typical in complex media, make the comparison of different MET studies difficult. As an example, natural sediments involve both an organic and an inorganic matrix that can highly vary depending on the location, season, or historical events such as microbial activity.^15^ Thus, METs require additional tools to detect changes happening in the bulk potential of complex media and correct possible inconsistencies derived from changes in the bulk potential while helping to standardize between experiments.

In anodic studies, a reducing environment and an oxidizing electrode are necessary. If the cathodic half-cell in the MFC is maintained constant (*e.g.*, using ferredoxin), changes in the anode affect the final power output. When the anode is in a more reductive environment the whole-cell potential increases and the current flows. However, if the oxygen coming from the cathode leaks inside the anode, the redox value of the anode environment increases, leading to power drops.^16,17^ Similarly, in sediment microbial fuel cell (SMFC) installations, events such as tides, waves or storms can dig up the anode from inside the sediment into the overlaying oxygenated water, causing the power output to stop. The bulk potential serves as a reference value to guide oxidation or reduction potentials depending on the MET application (power source *vs.* synthesis). Thus, monitoring the bulk potential is an important piece of information for MET operation. The use of an auxiliary electrode (AE), incorporated inside a microbial electrochemical cell, monitors the open circuit (OC) potential as a measure of the bulk electrolyte potential. The concept of AE was first introduced as a ‘Pin electrode’ in a MFC half-cell and further developed to assess its application in sensing, power modulation and electrosynthesis.^18,19^ Additionally, MFC as a smart sensor was successfully demonstrated using an AE without disturbing internal redox system.^20^

The microbial growth on the electrode surface changes in electrochemical characteristics of the electrode^21^ by mediating the transfer of electrons (electroactive) or electrically isolating the electrode from the environment (non-electroactive). It has been demonstrated that electroactive microorganisms can be grown on the anodic or working electrode surface by applying a controlled anode potential ranging from −58 to 680 mV *vs.* Ag/AgCl reference electrode.^22,23^ In a previous report, poising or polarization of working electrode at +200 mV *vs.* Ag/AgCl reference electrode, not only reduce the start-up time of MECs but also results in increased current production.^23^ In this study, the WE was polarized at +200 mV *vs.* RE. By using the integrated non-polarized AE, this paper shows how it can be used to compare electrochemical techniques and clearly distinguish the effect that continuous polarization can have on electrochemical parameters over time for sediment clean-up. For this reason, a new type of reactor was developed to showcase the utilization of the AE in a four-electrode system (three-electrode cell + AE). Information on the construction, behaviour and variability of the do-it-yourself (DIY) reactors is provided to promote the use of AE in other MET experiments. Future improvements of the system are suggested as well as ways to further reduce the variability of METs performance in treating complex substrates.

## 2 Methods and materials

### 2.1 Inoculum and media

Sediment was collected from the Lough Atalia estuarine lagoon (53.28′N, −9.03′W) in Galway (Ireland), 2 meters from the north–east shore. This location was identified as a potential location for SMFC installation due to sediment accumulation, eutrophication and mesohaline conditions.^24^ Sediment samples were harvested from the mud located immediately below the vegetation level. The sediment was sieved (2 mm grid) to eliminate macrofauna, stones and plant debris followed by centrifugation at 10 000 rpm for 6 minutes and the supernatant (pore water) was analysed [Table 1, see ESI†] and discarded. The centrifuged sediment was stored at −18 °C until further use in the experiment. Each reactor was inoculated with 10 g of sediment.

Artificial seawater (ASW) was used as the electrolyte in two different concentrations resulting in salinities of 50 and 12 mS cm^−1^ and pH 8.5 and 8.2, respectively [Table 1, see ESI†]. Propionic acid was used as an additional carbon source at different stages of the experiment. Two different solutions were produced for the two assessed salinity levels. A stock of 1 g L^−1^ and 10 g L^−1^ were prepared for each salinity level and kept refrigerated (4 °C) until use.

### 2.2 Reactor set-up

**Fig. 1:**
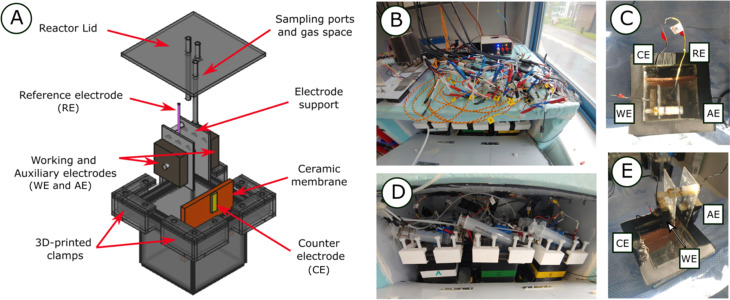
Cubic reactor 3D model and schematic showing the WE and AE placed in the electrode holder, CE in yellow, ceramic membrane in orange, 3D-printed clams and RE between the WE and AE (A). Photograph of the OpenTCC and the wiring of the 6 WE, 6 AE, 6 CE and 6 RE (B). Photograph of the top of one cubic reactor setup with labels on the WE, CE, AE and RE (C). Photograph of the six cubic reactors inside the OpenTCC, note the syringes and gas flushing system (D). Photograph of a side view of the cubic reactor showing the electrode holder (E).

A novel reactor type was 3D printed (Ultimaker 3, Utrecht, Netherlands) with polylactic acid (PLA) plastic on a cubic frame (10 cm) with an additional clamping square [Fig. 1A]. The interior of the cube was covered with acrylic sheets (1 mm thickness). A cubic geometry was selected to optimize the space used by each reactor. A total of four electrodes were installed including a three electrode system with working electrode (WE), CE and reference electrode (RE) as well as the previously described open-circuit AE. The AE and WE were kept in a symmetric configuration with equal distances between them (5 cm) with the help of an acrylic electrode support [Fig. 1A and E]. WE and AE were composed of graphite plates (5 × 5 × 1.5 cm) drilled in the middle in which a nylon screw was inserted. The acrylic support included two nylon screws (M8) that helped to keep the electrodes in place and at constant distance between each other. A Ti wire (1 mm *Ø*) was utilized as current collector and joined to the electrodes by exercising pressure with a nylon nut until the resistance was below 4 Ω. The graphite plates were previously polished with P80 SiC sandpaper to clean and obtain uniform surfaces. An acrylic lid was installed and clamped on the top using four 3D-printed PLA U-shaped structures [Fig. 1A] and nylon screws (M8). Neoprene gaskets were utilized to seal the space between the reactor frame and lid.

Three ports were installed in the lid including two sampling ports for the WE and CE chambers and a gas space port [Fig. 1A]. The WE and CE sampling ports used an acrylic tube that descended inside the reactor until 1 cm from the bottom, while the gas space port was kept at the same height of the lit. The WE and CE chambers were kept separated using unglazed ceramic membranes (Jodhpur, India) as described previously^25^ and kept at fixed distance by using silicone sealant. The ceramic membrane was confirmed to not have leaks.

The reactors were filled to keep 2 cm of gas space for each reactor and the final electrolyte volume was 430 mL for the WE chamber and 160 mL for the CE chamber. Silver/silver chloride reference electrodes were prepared and tested before use to keep homogeneous reference values throughout the experiment and placed between WE and AE inside the electrode holder [Fig. 1C and E]. The CEs consisted of platinized titanium mesh and titanium wire providing enough surface to drive counter electron reactions resulting from the WE constant polarization and the AE and WE electrochemical analyses (Section 3.1) and placed at the other side of the ceramic membrane [Fig. 1C and E].

### 2.3 Experimental design

**Fig. 2:**
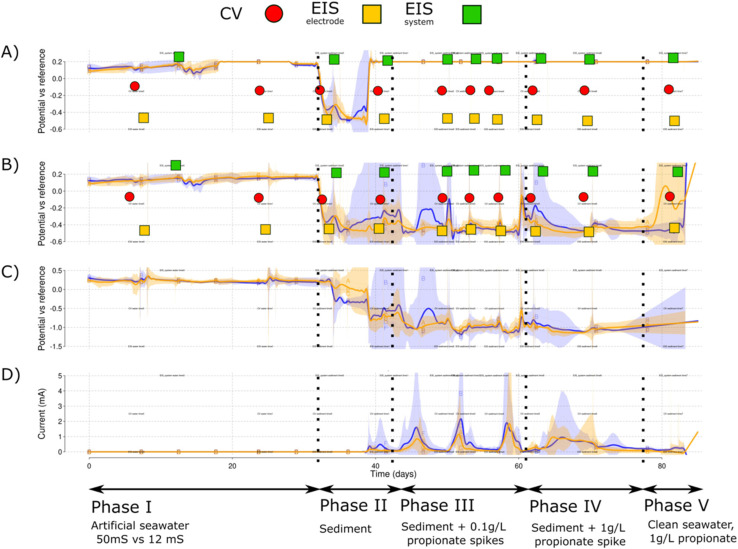
Simplified curves for each of the cubic reactor phases. Marked with red circles, the CV for WE and AE, yellow squares for the electrode EIS analyses and green squares for the whole cell EIS. The graphs show the mean values for the potential *vs.* RE in each of the salinity levels for the WE (A), AE (B) and CE (C) and finally the current production (D). Shadowed areas indicate the CI for each of the parameters measured. A blue color is used for the low salinity and orange for the high salinity level of the triplicate reactors.

A total of six cubic reactors were built and kept at constant temperature inside a temperature-controlled chamber at 30 °C [Fig. 1D]. Reactors were labelled from A to F with A, C and E the high salinity (50 mS cm^−1^) and B, D and F the low salinity (12 mS cm^−1^) reactors. Continuous stirring (120 rpm) was applied for each of the reactors with a magnetic bar. The wiring of the 24 electrodes (4 × 6 reactors) was facilitated by providing connection strips on top of the temperature-controlled chamber [Fig. 1B]. The connection strips were then used to connect the electrode probes to the different available channels of a VMP3 potentiostat (Biologic, Seyssinet-Pariset, France) and a Picolog ADC-24 data-logger (PicoTech, Cambridgeshire, UK). A constant polarization potential of 0.2 V *vs.* RE was applied to the WE of each reactor. The experiment was conducted in five phases: initial start-up with ASW (Phase I), addition of sediment (Phase II), low concentration propionate spikes (Phase III), high concentration propionate spike (Phase IV) and a final phase with fresh ASW and high propionate concentration (Phase V) [Fig. 2].

Cyclic voltammetry (CV) and electrochemical impedance spectroscopy (EIS) were obtained spaced over time (every 2–3 days) throughout the length of the experiment for both AE and WE. Additionally, EIS of the whole system (WE/AE to CE) were also produced. CVs were performed at different scan rates 100, 50, 10, 5, 1 mV s^−1^ to assess differences between electrolyte and surface level electrochemical responses.^26^ The EIS was executed by applying a 10 mV peak-to-peak excitation single sinusoidal wave from 10 Hz to 10 kHz centred at a constant potential of 0.2 V.

### 2.4 Data analysis and treatment

The WE, AE, CE potentials and the current production from the WE were monitored and plotted over time for the different phases of the experiment. A total of 50 CVs (10 timepoints × 5 scan rates) and 9 EIS were obtained during the 90-day experiment. Due to the high amount of CV analyses obtained, an automated programming script was developed. Oxidative and reductive curves were isolated and plotted together for each of the scan rates under evaluation. At the end, only the mean and 95% CI (confidence intervals) were plotted for each of the timepoints of analysis. Similarly, EIS were obtained and plotted both in Nyquist and Bode plot form. Due to the noisy results obtained from the EIS of the electrodes, a linear fit was obtained for the region of interest of the electrode Nyquist plot (between 1–8 and 1–6 for the real and imaginary parts of the Nyquist plot, respectively). All plots and continuous CI calculations were conducted using the R programming platform. The electrolyte quality characterization was compared using a two-tailed *t*-test using Excel (Microsoft 365 version 2110).

### 2.5 Chemical analyses

The electrolyte composition of the WE chamber was analysed for total organic carbon (TOC) using a TOC analyser (Shimadzu TOC-L series, Kyoto, Japan) and tested for pH and conductivity using a pH meter (Mettler Toledo, FiveEasy F20, pH probe Mettler Toledo, LE438 PH, Greifensee, Switzerland) and conductivity meter (Horiba LAQUAtwin EC-22, Kyoto, Japan). Ammonium, conductivity, pH, iron, phosphate and sulfate were analysed using Gallery Plus discrete analyzer (Thermo Scientific, Waltham, United States) as described earlier.^27^ Elemental composition was obtained using an inductive coupled plasma instrument (Agilent ICP-OES 5110, Santaclara, USA) whereas ions were obtained using ion chromatography (Dionex Aquion, Thermo Scientific, Waltham, United States) with an IonPac AS14A 4 × 250 mm column coupled with an AG14A 4 × 50 mm guard column and sodium carbonate 3.03 mM/sodium bicarbonate 0.97 mM eluent at a flow rate of 1 mL min^−1^ as described previously.^27^ VFA concentrations were analysed by high performance liquid chromatography (HPLC 1260 Infinity II, Agilent, Santaclara, USA) equipped with a Hi-Plex H column held at 60 °C and a refractive index detector held at 55 °C with H_2_SO_4_ (5 mM) as the mobile phase at a flow rate of 0.7 mL min^−1^ as reported earlier.^28^

## 3 Results

### 3.1 Experimental phases

The experiment ran over 90 days. The system was continuously monitored by measuring each of the electrode potentials (WE, AE and CE) *vs.* RE. During open-circuit of the WE, both AE and WE behaved similar to each other. This can be observed for days 0 to 18 and 30 to 38 days [Fig. 2A and B].

**Fig. 3:**
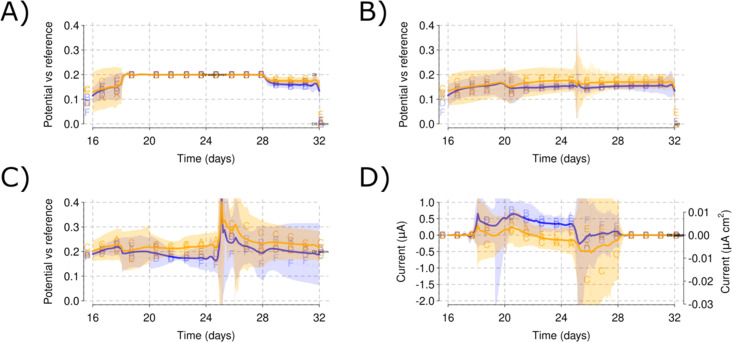
Cubic reactor artificial seawater monitoring during Phase I polarization plotting the mean values for the potential *vs.* RE in each of the salinity levels for WE (A), AE (B) and CE (C) and the current production at the WE (D). Shadowed areas indicate the CI for each of the parameters measured. A blue color is used for the low salinity reactors and orange for the high salinity reactors.

During the open circuit part of Phase I, a mean WE potential of 0.17 V *vs.* RE was obtained [Fig. 2A and B]. Between days 18 and 25, a polarization of 0.2 V *vs.* RE was applied to the WE [Fig. 3A] to provide an anode potential suitable to induce microbial film growth. The polarization generated a background current lower than 1 μA [Fig. 3D]. The AE showed a stable potential that remained below 0.2 V at both salinities [Fig. 3B]. However, an increased pull toward 0.2 V was found for the higher salinity level [Fig. 3]. Slight increases in current and lower AE potentials were observed when nitrogen flushing was applied on day 20 [Fig. 3B]. No redox peaks were evident in the CV response for WE or AE.

### 3.2 Initial sediment addition

**Fig. 4:**
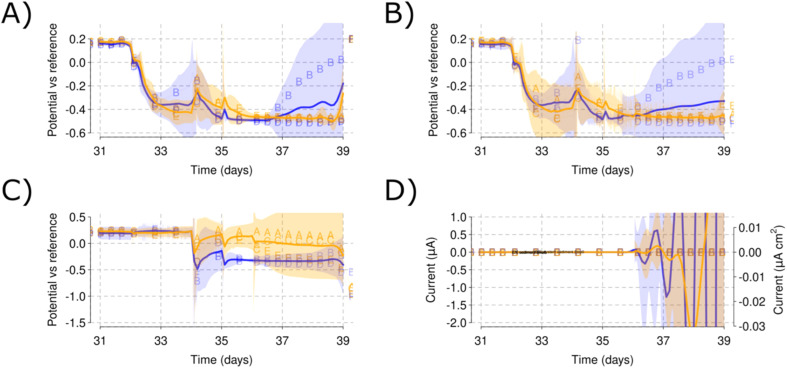
Initial open circuit stabilization with the sediment in Phase II. WE (A) and AE (B) show identical behaviour with a decrease in their potential *vs.* RE. Note how Reactor B (low salinity) separates itself while increasing the CI of the low salinity reactors. CE (C) similarly changes towards more reducing conditions. The current (D) shows a strange behaviour due to the low scale of the axis and the effect of the posterior polarization in the smoothing function. Color blue is used for the low salinity and color orange for the high salinity triplicate reactors.

**Fig. 5:**
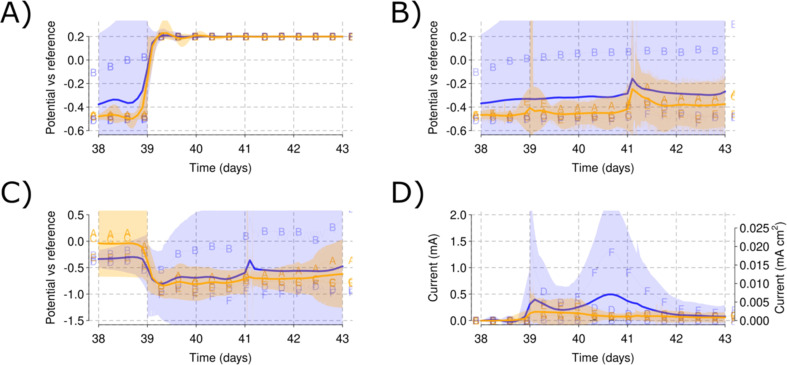
Initial polarization with sediment and monitoring of each electrode potential during Phase II. WE (A) reaches the applied 0.2 V *vs.* RE and maintains that steady. AE (B) remains at −0.5 V *vs.* RE in all the reactors except for Reactor B (low salinity) that reached up to 0.1 V *vs.* RE. CE (C) values reach under −0.5 V *vs.* RE except for Reactor B, that did not require that much current to get polarized to 0.2 V due to the air leak. Current production (D) shows a high initial peak concomitant with the beginning of the polarization and a second peak for Reactor F (low salinity). Shadowed areas indicate the CI for each of the parameters measured. A blue color is used for the low salinity and orange color for the high salinity triplicate reactors.

**Fig. 6:**
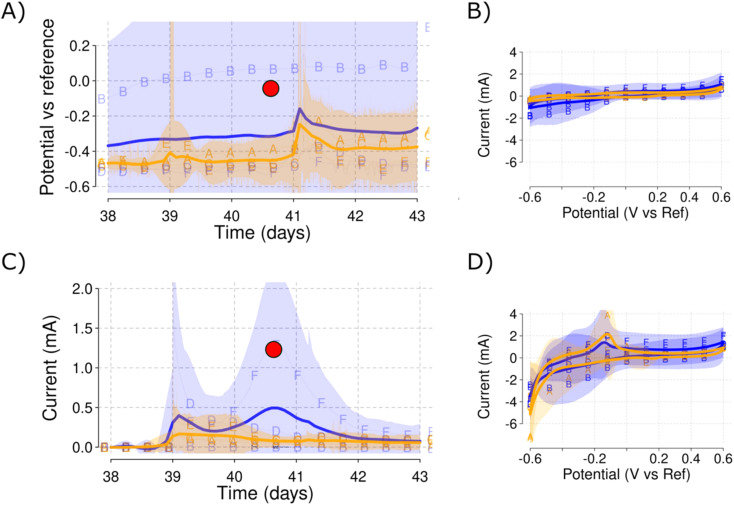
Auxiliary electrode potential (A), CV of the AE (B), current production (C) and CV of the WE (D). CVs for AE do not show any redox peak, (B) while the CVs for WE show a distinctive peak at −0.1 V *vs.* RE (D). Red dots show the time of the CV analyses. Shadowed areas indicate the CI for each of the parameters measured. A blue color is used for the low salinity and orange for the high salinity level.

**Fig. 7:**
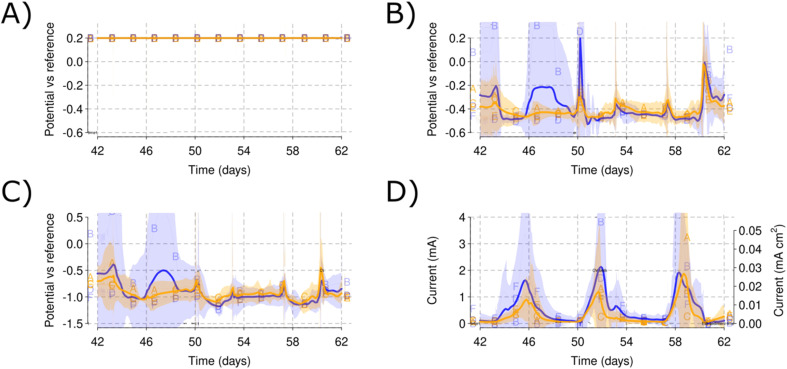
WE (A) potential monitoring during the polarizaion of Phase III. AE (B) potential showing the recovery of Reactor B (low salinity) to reduced conditions. CE (C) showing the return of Reactor B to the lower reducing potentials. Current production (D) for the three different low propionate concentration (0.1 g L^−1^) spikes. Note after recovery of the reduced conditions in Reactor B, the current peak of Reactor B with the propionate reaches up to 4 mA. Shadowed areas indicate the CI for each of the parameters measured. A blue color is used for the low salinity and orange for the high salinity level.

When the sediment was added to each of the reactors operating at open circuit, the AE potential progressively changed towards lower values and stabilized around −0.5 V *vs.* RE. However, Reactor B (low salinity) did not maintain the low potential and the AE potential increased towards the values observed during Phase I [Fig. 4B]. After stabilization of the open circuit potentials following sediment addition (by day 39), the WE potential was set again at 0.2 V *vs.* the RE. The current increased rapidly after the initial polarization and then decreased. However, posterior (day 40) currents up to 1.2 mA for Reactor F increased the mean of the low salinity reactors [Fig. 5 and 6]. This behaviour was missing for the other reactors which causes a large CI for the low salinity reactors further intensified by the low currents in Reactor B [Fig. 7D]. This can also be observed by the large CI for CE [Fig. 7C] due to the CEs being driven to reducing values except for Reactor B.

While the variability for the current production was large, the polarization imposed in the presence of sediment in Phase II developed a distinctive redox peak at −0.1 V *vs.* RE. This redox peak was not visible in the CVs recorded using the AE [Fig. 6C] and was only possible to detect in the CVs recorded for the WE in the low-speed CV scan rate of 1 mV s^−1^ [Section 3.7]. The redox peak is clearly visible in both high and low salinity electrolyte, but the high salinity reactors develop a higher current peak due to the important contribution of the peak in Reactor A [Fig. 6D]. Note how the distinctive redox peak presents higher mean currents for the high salinity reactors and particularly in Reactor A, while the continuous mean current production is higher for the low salinity and Reactor F.

### 3.3 Propionate spikes

After the initial polarization, spikes of propionate at low concentration (0.1 g L^−1^) were introduced while maintaining the WE polarization [Fig. 5A], which generated current for each of the reactors. During this process, Reactor B recovered reduced conditions by simply tightening its lid which can be observed by the values of the AE between days 46 and 50 [Fig. 5B]. Current production up to 4 mA was obtained for Reactor B (low salinity) and Reactor A (high salinity) for the 2nd and 3rd spike, respectively [Fig. 5D]. The CE and AE values for the low salinity reactors seem to run in parallel because of the influence of the poorly closed lid on the environment of Reactor B [Fig. 5C].

After assessing the low propionate concentration, a 1 g per L spike was provided to each of the reactors (day 61). The AE potential increased in Reactor B due to a possible leak of air during handling of the reactor but recovered back to reducing redox environments on day 65 [Fig. 7B]. The CE kept a mean value of −1 V *vs.* RE, driving the reducing reactions required for the WE polarization [Fig. 7A and C]. The current did not increase to a level over the previously obtained currents at the low propionate concentration, showing a maximum of 2.4 mA for Reactor D [Fig. 7D], which also showed the lowest AE potential [Fig. 7B].

**Fig. 8:**
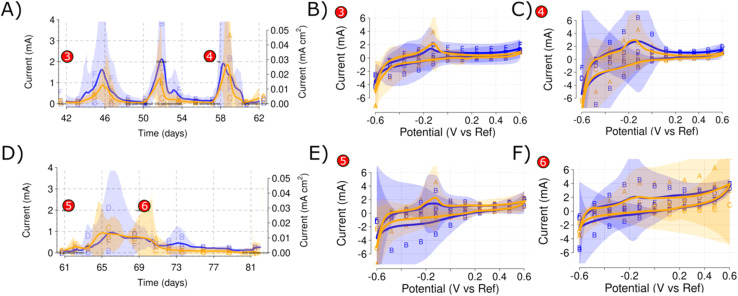
Comparison of current production between the 0.1 g L^−1^ (A) and 1 g L^−1^ propionate (D) spikes. The selected CVs at different timepoints showcase how the high propionate concentrations result in a lower prevalence of the redox peak at −0.1 V *vs.* RE. CV number 3 (B) shows the previously described redox peak, formed during the polarization with sediment. CV number 4 (C) shows the redox peak after two low propionate concentration spikes. CV number 5 (E) shows the redox peak after the third low propionate concentration spike. CV number 6 (F) shows the redox peak disappearing after the high propionate concentration spike.

While mean current values reached up to 2 mA for both salinity levels in the low propionate concentration spikes, a mean current value of less than 1 mA was obtained on the high propionate concentration spike [Fig. 8A and D]. The CVs performed during this propionate spike show how the low propionate concentrations increased the redox peak for both salinity levels [Fig. 8C and E]. However, the high salinity level practically removed the redox peak that became part of a straight oxidation line [Fig. 8F]. The high redox peak for Reactors A and B corresponds well with the higher currents shown during the 3rd low propionate concentration spike [Fig. 5D and 8A].

### 3.4 CV speed effect

**Fig. 9:**
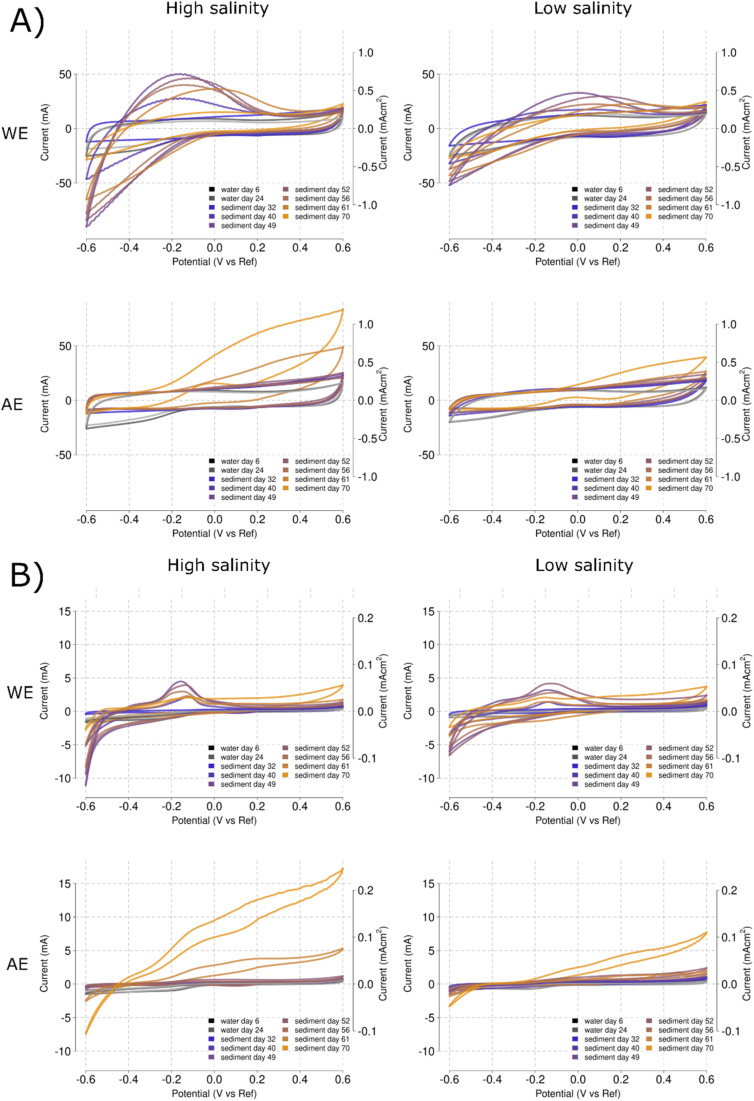
Mean CV fingerprints for each time point obtained at 100 mV s^−1^ (A) and 1 mV s^−1^ (B). For each speed, the measurements were performed at 50 mS cm^−1^ (high salinity) and 12 mS cm^−1^ (low salinity), salinities are indicated at the top of the graph. Fingerprints were overlaid over time for both the WE (top panels) and AE (bottom panels). Times are color-coded with a gradient from blue (32 days) to yellow (70 days). Time points before the sediment addition (day 6 and 24) are indicated in grey.

CV rates have a particular effect on the CV fingerprints. The high scan rate CVs in the WEs [Fig. 9A] indicate higher current means than the AE counterparts [Fig. 9A]. However, the CV curves do not center around a specific potential point and peaks oscillate between −0.2 and +0.2 V *vs.* RE. The CV of AEs do not show an intermediate peak and remain relatively flat except for oxidative currents at the higher potentials [Fig. 9A]. Slower scan rates define the redox peak and the value gets centered at −0.1 V *vs.* RE [Fig. 9B]. However, towards the end of the experiment, the CV fingerprint on day 70 resembles more those found in the AE, with increasing currents at increasing oxidative potentials [Fig. 9B]. Note how the last CVs (days 61 and 70) show higher currents in the AE than in the WE [Fig. 9A and B].

### 3.5 Evolution of EIS of full cell and the electrodes over time

**Fig. 10:**
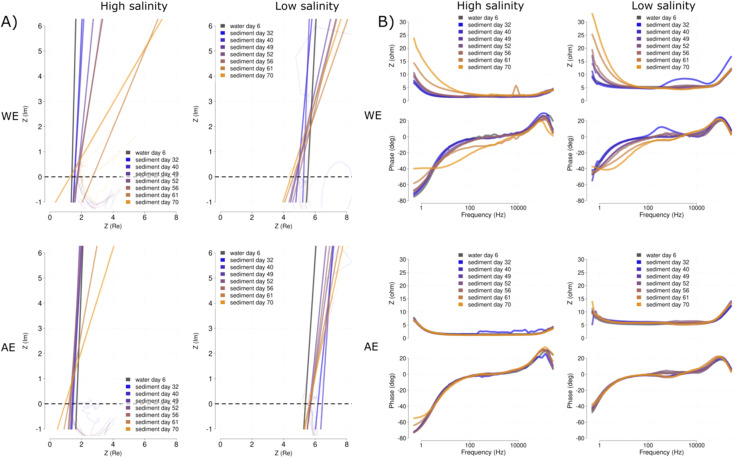
Mean EIS fingerprints of the electrode for each time point represented in the form of Nyquist (A) and Bode (B) plots. For each representation the measurements are shown for the 50 mS cm^−1^ (high salinity) and 12 mS cm^−1^ (low salinity), salinities are indicated at the top of the graph. Fingerprints were overlaid over time for both the WE (top panels) and AE (bottom panels). Times are color-coded with a gradient from blue (32 days) to yellow (72 days). Time point before the sediment addition (day 6) is indicated in grey.

The EIS of the electrodes showed differences when comparing WE and AE. Overtime, the EIS tended to show a lower slope for the WE in both salinities [Fig. 10]. This change in slope was more marked for the high salinity. However, other parameters obtained from the Nyquist plot such as resistance and capacitance of the electrode were not possible to be obtained as the response showed a chaotic behaviour. However, the modulus values (*y*-axis) in the Bode plot tend to increase over time for the WE [Fig. 10], while the modulus values for AE kept steady [Fig. 10]. Similarly, the phase values in the Bode plots decrease for the frequencies between 10 and 1000 Hz for the WE while maintaining the same range in the AE. These patterns seem to affect more the low salinity reactors than those with the higher salinity. The EIS with higher propionate concentrations (days 61 and 70) seem to accelerate the changes described above [days 61 and 70] and also affect the AE Nyquist results [Fig. 10] and the alpha and modulus values of the high and low salinity reactors, respectively [Fig. 10].

**Fig. 11:**
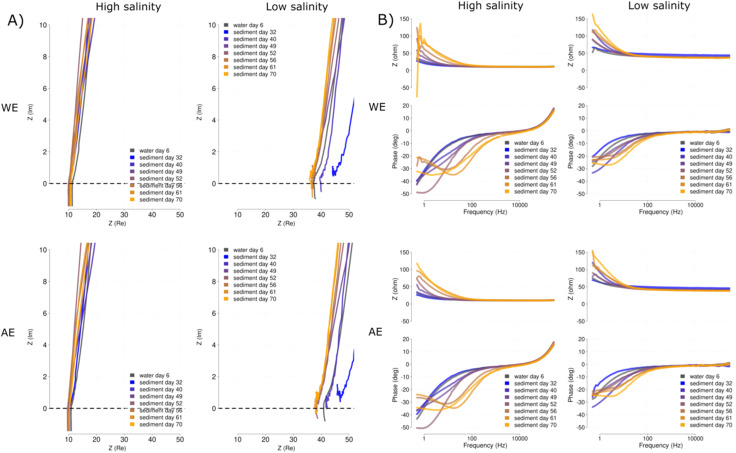
Mean EIS fingerprints of the whole system for each time point represented in the form of Nyquist (A) and Bode (B) plots. For each representation the measurements are shown for the 50 mS cm^−1^ (high salinity) and 12 mS cm^−1^ (low salinity), salinities are indicated at the top of the graph. Fingerprints were overlaid over time for both the WE (top panels) and AE (bottom panels). Times are color-coded with a gradient from blue (32 days) to yellow (72 days). Time point before the sediment addition (day 6) is indicated in grey.

The EIS of the whole system does not seem to draw as clear differences as those found for the single electrodes. The Nyquist plots show similar behaviour for both WE and AE, with a slightly lower impedance for the WE in the low salinity reactors [Fig. 11]. The lower impedance in the high salinity reactors correspond to the lower impedance found during testing of the ceramic membranes (see Section 3.2). This is also visualized in the Bode plots, for which the alpha changes [bottom axis in Fig. 11] are in a larger range (*α* −50) for the high salinity than for the low salinity (*α* −30) reactors.

## 4 Discussion

### 4.1 Open circuit AE potential and monitoring for troubleshooting reactors

This study showed that the open circuit AE provides an additional control parameter for electrochemical polarisation techniques. CV and EIS analysis are sometimes difficult to interpret during initial screening of microbial electrochemical processes due to the large amount of variability and technical factors involved.^29^ In this study, the use of open circuit AE enabled that the changes to CV [Fig. 6 and 9] and EIS [Fig. 10] over time and conditions were clearly distinguishable.

The open circuit AE can be used to monitor the bulk ORP (oxidation–reduction potential) for each of the reactors. Small changes in the electrolyte composition during nitrogen flushing [Fig. 3] or changes caused by the sediment addition [Fig. 4] resulted in changes to the ORP as monitored by the AE potential values. The open circuit AE can, therefore, serve as a troubleshooting approach to detect oxygen intrusion into Reactor B [Fig. 3 and 6]. This presents a useful method to monitor electrolyte conditions in MFC and MET. It may represent a low-cost alternative to oxygen sensors, and could aid in alleviating, early on, the biases derived from reactor malfunctioning. As an example, Reactor B showed an increase in the AE potential that was traced to a leak in this reactor permitting ingress of air, which was solved by tightening the clamps of the reactor lid. The increased values of the AE potential correlated with its low current production that skewed the comparison between the low and high salinity reactors.

The ORP monitoring is a valuable addition to be considered in MET as it can be helpful in monitoring bulk potential changes, detecting anomalous behavior or correcting for the effect of polarization in electrochemical analyses. Thus, an AE can solve some of its challenges during screening and laboratory tests with different substrates (that generate different bulk potential values) and correct for the effect on current of variations in inoculum and microbial population dynamics that cause changes in the AE potential.

The AE kept a stable potential during Phase I polarization [Fig. 3]. This suggests the AE could be used as a new type of reference electrode. Thus, the open circuit AE could be a new method of applying potentiostatic METs if positive feedback on the WE–AE interaction is avoided. Keeping enough resistance between the WE and AE would then be necessary for this application. The high salinity reactors showed a slight increase in their potential [Fig. 3] for the utilized WE–AE distance (5 cm). Due to possible changes in electrolyte conductivity, additional controls should be included for the AE to be effective. Similarly, if there is oxygen intrusion, the AE would not be able to detect it because it still needs a constant electrochemical potential value to be compared.

### 4.2 Current production

#### 4.2.1 Non-faradaic initial response and current generation peaks

After stabilization of the open circuit potentials following sediment addition, the WE potential was set at 0.2 V *vs.* RE [Fig. 4]. This generated a current output in all reactors, which subsequently decreased and stabilized. This effect is clearly observed for the individual reactors rather than by plotting mean currents, due to the smoothing effect of the mean value of the triplicates. This initial peak-shaped response in current can be caused by an initial attraction of charge that was apparently not continued over time. This points to a non-faradaic attraction of the salts and other negative charged components inside the reactor.^30^ The variable response between the reactors was probably caused by small changes in the electrolyte and sediment concentration, but could also be derived from small changes between the WE surfaces. Due to the high-sensitivity of non-faradaic currents, understanding their response can also be important to detect variability between different electrode setups or discovering new factors involved in the electron transport mechanisms.

Reactor F, despite being a replicate under the same low-salinity conditions, showed a clear increase in current density following initial polarization, unlike reactor B, which maintained a low current (Fig. 4). This highlights the importance of replicates in METs, at least during screening experiments presenting different start-up times.^31^

#### 4.2.2 Propionate spikes

The low propionate concentration spikes resulted in a replicable response of the current for the different reactors at each salinity level [Fig. 8]. The mean current was around 2 mA per reactor and up to 4 mA maximum (0.05 mA cm^−1^) was obtained for both Reactors B (low salinity) and A (high salinity). For each low propionate concentration spike, it took approximately four days to recover the current to background levels. However, high concentrations of propionate did not reach similar values, with mean current production below 1 mA per reactor [Fig. 8D] and just about 2.5 mA for Reactor D [Fig. 8D]. The time required to return to background currents was about 10 days.

The supplied propionate was not completely oxidized in the high salinity spikes, with acetate concentrations increasing for the low salinity [Table 3, see ESI†]. This indicates that the currents obtained from the low propionate concentration spikes were not from pure electrochemical origin and there was a microbial process catalysing the electron transfer to the WE. It is possible that the high propionate concentrations had a toxic effect on the microbial communities present inside the reactors and inhibited the previously developed electroactive microorganisms. This could be checked by comparing reactors with and without the propionate spikes and sampling the electrode surfaces to characterise the microbial community, *e.g.* by fluorescence *in situ* hybridization (FISH).^32^

### 4.3 Electrochemical analyses

#### 4.3.1 Cyclic voltammetry

The biocatalysis of organic compounds deduced from the propionate spike currents was further confirmed using CV [Fig. 9]. The characteristic redox peak developed at the WE during the sediment addition [Fig. 6] was further defined during the low propionate concentration spikes and almost disappeared after the high propionate concentration spike [Fig. 8]. Slight variations between replicates were found that could be caused by the different response to the stress of the microbial communities present inside the reactors. Variation between replicates can completely distort the results and make interpretation unclear with only one measurement.^33^ However, the redox peak tended to disappear during the subsequent CVs [Fig. 9].

The comparison between AE and WE CVs shows completely different patterns highlighting again the use of open circuit AE to control, troubleshoot and interrogate system parameters. The fact that the last timepoints tends to present high oxidation currents in the AE might point to purely electrochemical effects and non-faradaic currents of attraction and repulsion of the charged, dissociated propionate. However, it could also be partially caused by the detachment of the redox intermediates from the WE into the electrolyte, that are object to the same effect of non-faradaic currents of attraction and repulsion described for the substrate propionate.

The apparent contradiction between current production and CV peak currents in Phase II, where high current production did not correspond with a higher CV redox peak [Fig. 4], disappeared when several CVs were obtained during propionate spikes [Fig. 9]. This highlights again the need of applying several CVs over time to learn from the system response. The possible changes in microbial activity and charge concentrations should tend to equalize over time if the same environment is provided. CV is a valuable technique to characterize METs, however, a single CV should be considered carefully if final conclusions are to be obtained. Additionally, CV cannot be applied over time without the risk of influencing the microbial communities.^34^ The range of potentials and speeds are important parameters that should be carefully planned, avoiding oxygen and hydrogen evolution that might damage surface attached microorganisms or alter the community composition. Therefore, in METs that use potentials close to the hydrogen generation potential (MES), the range of potentials during CVs should be restricted to avoid damages to the biofilm integrity caused by uncontrolled gas production.

#### 4.3.2 Electrode EIS

EIS is a sensitive analytical technique which is often used to characterize material properties.^35^ However, its use in MET is controversial due to the limitations caused by large surface electrodes and the high distances to the RE.^36^ The relatively small surface of the electrodes used in this experiment (70 cm^2^) is actually large from the perspective of electrochemical analyses where surfaces of 1 cm^2^ are common.^35^ Similarly, the relatively small distance from the WE and AE to the RE (1 to 4 cm) is undoubtedly large for electrochemical analysis standards that must be kept to the minimum, in the order of millimeters.^13^ This was probably the cause of the behavior observed during the electrode EIS [Fig. 10]. However, the clear differences in the diffusion slope show the effect that polarization had on the close distance of the electrode. This could be caused by the attraction of more negative charges on the polarized electrode and not by the effect of microbial growth.

Changes in resistance and capacitance are typical in MET electrodes during long-term experiments.^37^ They can be diagnosed using EIS and determine if changes are caused by membrane clogging^38,39^ or electrode passivation.^40,41^ However, electrode-related EIS require new solutions in order to improve the quality of the EIS results in METs. The use of a small electrode portion for the EIS could be a potential solution. The large surface of the electrodes typical in METs can completely change the CV and EIS fingerprint of the system due to material capacitance. During recording of a CV, many of the redox processes may remain hidden and during EIS recording, chaotic results can be produced, difficult to interpret. As shown in Fig. 9, this could be partially compensated by selecting slower scan rates. However, large surfaces could force to excessively low scan rates that can cause changes in the microbial communities adapting to the changing potentials. Therefore, using polarized small surface AE is necessary to standardize both CV and EIS analyses of METs.

### 4.4 Future perspectives of open-circuit auxiliary electrodes in microbial electrochemical systems

MET offer promising solutions for wastewater treatment, bioenergy production, and environmental remediation. However, several challenges still hinder their large-scale application. Key limitations include inconsistent electrochemical performance, fluctuations in redox conditions, low electron transfer efficiency, and difficulty with real-time monitoring and control.^42^ Biofilm stability in dynamic environments, such as river clean-up applications, remains a significant challenge, as external disturbances can disrupt microbial activity and electron transfer.^43^ Additionally, BES often suffer from oxygen intrusion, material degradation, and performance variability, reducing their long-term operational efficiency.^42^ The incorporation of an AE represents a significant advancement in BES and METs. As presented in this study, the AE can be utilized for real-time monitoring of bulk potential without interfering with redox reactions, facilitating enhanced system control, operational stability, and standardization in diverse applications.

Incorporation of AE in MET can be helpful in tracking bulk redox potential, allowing precise control over microbial electrocatalysis and contaminant degradation. The AE can be particularly useful for treating complex wastewater streams, such as textile effluents and industrial discharges, by stabilizing biofilm activity and optimizing electron transfer. Additionally, in anaerobic digestion-assisted METs, AE-based monitoring can optimize nutrient recovery and energy conversion.

The AE can support electrolyte monitoring in BESs, improving system efficiency and power output. It can be employed in SMFCs to maintain electrochemical stability by detecting shifts in bulk potential caused by environmental disturbances like tides or oxygen diffusion. In MESs, the AE can play a crucial role in maintaining optimal redox conditions for CO_2_ conversion into value-added products such as acetate and methane, making it an essential tool in bio-based chemical production. In agriculture and soil remediation, AE-assisted METs can be utilized for enhancing microbial electrochemical soil remediation, aiding in the degradation of agricultural pollutants. Additionally, it can be applied in electrochemical-assisted irrigation systems, optimizing soil redox conditions to enhance nutrient availability and water retention for sustainable farming practices.

Similarly, the AE can particularly be valuable in river and pond clean-up, where fluctuating environmental conditions pose challenges in bioelectrochemical control.^44^ Integrating AE technology into floating microbial fuel cells and bioelectrochemical reactors can facilitate the degradation of organic matter and removal of nitrates and phosphates, contributing to ecosyste restoration. Furthermore, AE-assisted adaptive process control can help stabilize electrochemical conditions despite variations in river flow, temperature, and oxygen levels, improving scalability for aquatic bioremediation.

The AE enhances electrochemical sensing by improving the accuracy of EIS and CV, reducing variability in bioelectrochemical research. It can also function as a low-cost alternative to oxygen sensors, detecting oxygen intrusion that may disrupt anaerobic processes. Additionally, AE-based monitoring improves ORP-based control strategies, ensuring optimized microbial metabolism for energy-efficient bioelectrochemical processes. Ieropoulos *et al.* (2018) employed AE in both anodic and cathodic compartment and observed an increment of 79% and 33% with anodic AE and cathodic AE, respectively.^18^ Furthermore, it was successfully demonstrated that AE can be employed as an innovative tool for sensing open circuit potential to monitor and control BESs real time.^18^

In industrial-scale applications, AE technology might play a critical role in scaling up BES-based treatment systems. Recently, Khandelwal *et al.* (2024) demonstrated application of algae-assisted MFCs in biogas upgradation.^45^ AE can support BES employed in biogas upgradation, CO_2_ sequestration and other electrochemical based carbon capture processes. Gajda *et al.* (2021) sucessfully demonstrated various innovative methods of *in situ* modulation for electrosynthesis using AE and concluded that AE-based sensing can also enhance renewable energy storage applications, particularly in microbial battery systems that convert organic waste into electrical energy.^19^

Despite its advantages, the practical implementation of AEs in large-scale METs still can face some challenges. Issues such as biofouling, material degradation, and electrolyte composition can impact long-term stability, requiring periodic recalibration. Additionally, integrating AE-based monitoring into real-world systems demands highly sensitive instrumentation capable of withstanding environmental fluctuations, particularly in river clean-up applications where flow rate, temperature, and oxygen levels vary significantly. Addressing these limitations through material innovation and system optimization is essential for widespread adoption.

By integrating AE technology into BESs, researchers and industries can enhance the stability, scalability, and efficiency of microbial electrochemical processes, paving the way for next-generation sustainable energy and environmental solutions.

## 5 Conclusions

The AE was shown to be useful for troubleshooting, bulk potential monitoring and control of electrochemical analyses in MET. The use of replicates and CVs at different timepoints helped to identify trends from the propionate spikes. The EIS analyses showed a decreased value of the diffusion slope for the WE, but this was probably caused by the attraction of negatively charged particles. The EIS obtained were noisy and thus require smaller electrodes surfaces. Similarly, the high variability in electrolyte composition indicated that continuous feeding should be preferred to analyse the effect of MET on the electrolyte quality. Overall, the open circuit AE could distinguish electrochemical fingerprints and its installation in METs, such as MFCs, is highly recommended.

## Author contributions

Carlos Sánchez: conceptualization, methodology, validation, investigation, writing – original draft; Amitap Khandelwal: data curation; writing – review & editing; Piet Lens: resources, writing – review & editing, visualization, supervision, project administration, funding acquisition.

## Conflicts of interest

The authors declare that they have no known competing financial interests or personal relationships that could have appeared to influence the work reported in this paper.

## Supplementary Material

RA-015-D5RA03133H-s001

## Data Availability

All data generated or analysed during this study are included in this published article and its ESI file.† Detailed data for this article can be found at https://aran.library.nuigalway.ie/bitstream/handle/10379/17191/Carlos_Sanchez_Thesis_NUIG.pdf?sequence=1.

## References

[cit1] Khandelwal A., Dhindhoria K., Dixit A., Chhabra M. (2021). Superiority of activated graphite/CuO composite electrode over Platinum based electrodes as cathode in algae assisted microbial fuel cell. Environ. Technol. Innovation.

[cit2] Khandelwal A., Vijay A., Dixit A., Chhabra M. (2018). Microbial fuel cell powered by lipid extracted algae: A promising system for algal lipids and power generation. Bioresour. Technol..

[cit3] Logan B. E., Rabaey K. (2013). Conversion of wastes into bioelectricity and chemicals by using microbial electrochemical technologies. Science.

[cit4] Khandelwal A., Chhabra M., Lens P. N. L. (2023). Integration of third generation biofuels with bio-electrochemical systems: Current status and future perspective. Front. Plant Sci..

[cit5] Khandelwal A., Lens P. (2023). Simultaneous removal of sulfide and bicarbonate from synthetic wastewater using an algae-assisted microbial fuel cell. Environ. Technol..

[cit6] Desmond-Le Quéméner E., Bridier A., Tian J. H., Madigou C., Bureau C., Qi Y., Bouchez T. (2019). Biorefinery for heterogeneous organic waste using microbial electrochemical technology. Bioresour. Technol..

[cit7] Aulenta F., Puig S., Harnisch F. (2018). Microbial electrochemical technologies: maturing but not mature. Microb. Biotechnol..

[cit8] Harnisch F., Rabaey K. (2012). The diversity of techniques to study electrochemically active biofilms highlights the need for standardization. ChemSusChem.

[cit9] Logan B. E. , Microbial Fuel Cells, 2011, pp. 641–665

[cit10] Rabaey K., Rodríguez J., Blackall L. L., Keller J., Gross P., Batstone D., Verstraete W., Nealson K. H. (2007). Microbial ecology meets electrochemistry: Electricity-driven and driving communities. ISME J..

[cit11] Butti S. K., Velvizhi G., Sulonen M. L. K., Haavisto J. M., Oguz Koroglu E., Yusuf Cetinkaya A., Singh S., Arya D., Annie Modestra J., Vamsi Krishna K., Verma A., Ozkaya B., Lakaniemi A. M., Puhakka J. A., Venkata Mohan S. (2016). Microbial electrochemical technologies with the perspective of harnessing bioenergy: Maneuvering towards upscaling. Renewable Sustainable Energy Rev..

[cit12] Koch C., Korth B., Harnisch F. (2018). Microbial ecology-based engineering of Microbial Electrochemical Technologies. Microb. Biotechnol..

[cit13] Allen J. and Bard Larry R. F., Electrochemical Methods Fundamentals And Applications, 2000, pp. 117–157

[cit14] White H. K., Reimers C. E., Cordes E. E., Dilly G. F., Girguis P. R. (2009). Quantitative population dynamics of microbial communities in plankton-fed microbial fuel cells. ISME J..

[cit15] Zhang M., Wu Z., Sun Q., Ding Y., Ding Z., Sun L. (2019). Response of chemical properties, microbial community structure and functional genes abundance to seasonal variations and human disturbance in Nanfei River sediments. Ecotoxicol. Environ. Saf..

[cit16] Logan B. E., Hamelers B., Rozendal R., Schröder U., Keller J., Freguia S., Aelterman P., Verstraete W., Rabaey K. (2006). Microbial fuel cells: Methodology and technology. Environ. Sci. Technol..

[cit17] Neethu B. , Khandelwal A., Ghangrekar M. M., Ihjas K. and Swaminathan J., Microbial Fuel Cells-Challenges for Commercialization and How They Can Be Addressed, Elsevier Inc., 2022, pp. 393–418

[cit18] Ieropoulos I. A., You J., Gajda I., Greenman J. (2018). A New Method for Modulation, Control and Power Boosting in Microbial Fuel Cells. Fuel Cells.

[cit19] Gajda I., You J., Mendis B. A., Greenman J., Ieropoulos I. A. (2021). Electrosynthesis, modulation, and self-driven electroseparation in microbial fuel cells. iScience.

[cit20] Ieropoulos I. and Greenman J., Microbial fuel cell, method of controlling and measuring the redox potential difference of the fuel cell, WO2016120641A1, WIPO (PCT), 2016

[cit21] Jung S., Mench M. M., Regan J. M. (2011). Impedance characteristics and polarization behavior of a microbial fuel cell in response to short-term changes in medium pH. Environ. Sci. Technol..

[cit22] Kim B. H., Kim H. J., Hyun M. S., Park D. H. (1999). Direct electrode reaction of Fe (iii) reducing bacterium, Shewanella putrefacience. J. Microbiol. Biotechnol..

[cit23] Wang X., Feng Y., Ren N., Wang H., Lee H., Li N., Zhao Q. (2009). Accelerated start-up of two-chambered microbial fuel cells: Effect of anodic positive poised potential. Electrochim. Acta.

[cit24] National Parks and Wildlife Service, Special Area of Conservation: 000268 – Galway Bay Complex SAC, Government of Ireland, https://www.npws.ie/protected-sites/sac/000268

[cit25] Khandelwal A., Chhabra M., Yadav P. (2020). Performance evaluation of algae assisted microbial fuel cell under outdoor conditions. Bioresour. Technol..

[cit26] Richter H., Nevin K. P., Jia H., Lowy D. A., Lovley D. R., Tender L. M. (2009). Cyclic voltammetry of biofilms of wild type and mutant Geobacter sulfurreducens on fuel cell anodes indicates possible roles of OmcB, OmcZ, type IV pili, and protons in extracellular electron transfer. Energy Environ. Sci..

[cit27] Carboni M. F., Florentino A. P., Costa R., Zhan X., Lens P. (2021). Enrichment of autotrophic denitrifiers from anaerobic sludge using sulfurous electron donors. Front. Microbiol..

[cit28] Dessì P., Asunis F., Ravishankar H., Cocco F. G., Gioannis G. D., Muntoni A., Lens P. N. L. (2020). Fermentative hydrogen production from cheese whey with in-line, concentration gradient-driven butyric acid extraction. Int. J. Hydrogen Energy.

[cit29] Harnisch F., Freguia S. (2012). A basic tutorial on cyclic voltammetry for the investigation of electroactive microbial biofilms. Chem.–Asian J..

[cit30] Cobb S. J., Macpherson J. V. (2019). Enhancing Square Wave Voltammetry Measurements *via* Electrochemical Analysis of the Non-Faradaic Potential Window. Anal. Chem..

[cit31] Ortiz-Medina J. F., Call D. F. (2019). Electrochemical and Microbiological Characterization of Bioanode Communities Exhibiting Variable Levels of Startup Activity. Front. Energy Res..

[cit32] Chung K., Okabe S. (2009). Continuous power generation and microbial community structure of the anode biofilms in a three-stage microbial fuel cell system. Appl. Microbiol. Biotechnol..

[cit33] Ruiz Y., Baeza J. A., Montpart N., Moral-Vico J., Baeza M., Guisasola A. (2020). Repeatability of low scan rate cyclic voltammetry in bioelectrochemical systems and effects on their performance. J. Chem. Technol. Biotechnol..

[cit34] Smit S. M. d, Buisman C. J. N., Bitter J. H., Strik D. P. B. T. B. (2021). Cyclic Voltammetry is Invasive on Microbial Electrosynthesis. ChemElectroChem.

[cit35] Jorcin J. B., Orazem M. E., Pébère N., Tribollet B. (2006). CPE analysis by local electrochemical impedance spectroscopy. Electrochim. Acta.

[cit36] Dominguez-Benetton X., Sevda S., Vanbroekhoven K., Pant D. (2012). The accurate use of impedance analysis for the study of microbial electrochemical systems. Chem. Soc. Rev..

[cit37] Borole A. P., Aaron D., Hamilton C. Y., Tsouris C. (2010). Understanding long-term changes in microbial fuel cell performance using electrochemical impedance spectroscopy. Environ. Sci. Technol..

[cit38] Manohar A. K., Bretschger O., Nealson K. H., Mansfeld F. (2008). The use of electrochemical impedance spectroscopy (EIS) in the evaluation of the electrochemical properties of a microbial fuel cell. Bioelectrochemistry.

[cit39] Jing Y., Guo L., Chaplin B. P. (2016). Electrochemical impedance spectroscopy study of membrane fouling and electrochemical regeneration at a sub-stoichiometric TiO2 reactive electrochemical membrane. J. Memb. Sci..

[cit40] Mansfeld F., Lin S., Kim S., Shih H. (1988). Electrochemical impedance spectroscopy as a monitoring tool for passivation and localized Corrosion of aluminum alloys. Mater. Corros..

[cit41] Martinent A., Gorrec B. L., Montella C., Yazami R. (2001). Three-electrode button cell for EIS investigation of graphite electrode. J. Power Sources.

[cit42] Khandelwal A., Swaminathan J., Mangal A., Ghoroi C., Lens P. N. L. (2024). Comparing efficacy of anodic and cathodic chambers in a low-cost algae-assisted microbial fuel cell for textile wastewater remediation. Process Saf. Environ. Prot..

[cit43] Gupta S., Patro A., Mittal Y., Dwivedi S., Saket P., Panja R., Saeed T., Martínez F., Yadav A. K. (2023). The race between classical microbial fuel cells, sediment-microbial fuel cells, plant-microbial fuel cells, and constructed wetlands-microbial fuel cells: Applications and technology readiness level. Sci. Total Environ..

[cit44] Miwornunyuie N., Ugochukwu G., Hunter J. (2025). Evolutionary trends and development of constructed wetland coupled microbial fuel cell: A decade of development. J. Environ. Manage..

[cit45] Khandelwal A., Swaminathan J., Nolan S., Lens P. N. L. (2024). Coupling Biogas Upgradation and Dairy Wastewater Treatment for Simultaneous Carbon Capture and Bioelectricity Generation Using an Algae-Assisted Microbial Fuel Cell. ACS Sustain. Chem. Eng..

