# Supplementary material for: In situ electrochemical monitoring with an open circuit auxiliary electrode in microbial electrochemical cells treating sediments
Source: RSC Adv. 2025 Jun 25;15(27):21568–81. doi: 10.1039/d5ra03133h (PMC12188640; doi:10.1039/d5ra03133h)
Supplement: RA-015-D5RA03133H-s001 [file RA-015-D5RA03133H-s001.pdf]

Supplementary Information (SI) for RSC Advances.
This journal is © The Royal Society of Chemistry 2025

**Supplementary information**

In Situ Electrochemical Monitoring with an Open Circuit Auxiliary Electrode in Microbial Electrochemical Cells treating sediments

Author names and affiliations:

Carlos Sánchez[1], Amitap Khandelwal[1], and Piet N. L. Lens[1*]

1. Ryan Institute, School of Natural Sciences, University of Ireland, Galway, University Road, H91 TK33, Galway, Ireland.

* Corresponding author.

The name of the corresponding author, address, and e-mail address:

Piet N. L. Lens, Professor

Ryan Institute, School of Natural Sciences

University of Ireland, Galway,

University Road, H91 TK33

E-mail: piet.lens@universityofgalway.ie

**Electrolyte characterization**

Differences in the electrolyte composition between reactors were obtained between the end of Phase III and the end of Phase IV for the high [Table 2] and low salinity [Table 3] reactors. Differences between these two timepoints were more acute for the high salinity treatment, showing significant differences ($p \leq 0.05$) for ammonia, iron, pH, barium and acetic acid with all values tending to increase except the pH that tended towards a more acidic environment [Table 3]. The low salinity reactors, however, showed only a significant change ($p \leq 0.05$) in the ammonium concentration that tended to increase [Table 2]. Overall, the variability between the reactors tended to increase over time for all the analysed components with some exceptions [Tables 2 and 3].

While the sampling of the electrolyte was limited to the time before and after the propionate spikes, more changes in the electrolyte composition of the low salinity treatment were observed (Tables 2 and 3). The low salinity makes conductivity changes more significant. Similarly, the lower salinity showed a significant drop in pH after reactions [Table 2], probably due to a lower buffering capacity than the high salinity medium. The increase in ammonium concentrations was even more apparent, which was most probably slowly released from the sediment matrix and was likely stimulated by the polarization and propionate additions. However, due to the absence of a non-MET experimental control, it is difficult to consider if this release of ammonium was stimulated in the MET or just due to the natural decomposition of the sediment. Acetate and propionate were not detected in the high salinity reactors, (Table 2), but this could be caused by analytical errors as salinity is known to cause issues with HPLC analysis[1]. Additionally, the high variability found between the reactors also propagates large differences in statistical analyses. This is difficult to solve in batch reactors due to feedback processes caused by microbial activity and the effect of redox on MET performance. Therefore, improved reactor setups with continuous flow need to be developed for future studies that focus on more detailed analyses of the effect of MET operational parameters on the electrolyte composition.

Reference

1. Dulaquais, G.; Breitenstein, J.; Waeles, M.; Marsac, R.; Riso, R. Measuring dissolved organic matter in estuarine and marine waters: Size-exclusion chromatography with various detection methods. Environ. Chem. **2018**, *15*(7), 436–449.

**Table 1S:** Electrolyte composition of the artificial seawater at two different concentrations and the composition of the sediment pore water for the same parameters.

| Parameter | Unit | Sediment pore-water | Artificial Seawater (12 mS $cm^{-1}$) | Artificial Seawater (50 mS $cm^{-1}$) |
|---|---|---|---|---|
| Amonium | ppm | 62.50 | 27.23 | 15.02 |
| Conductivity | mS $cm^{-1}$ | 14.88 | 13.00 | 58.06 |
| Iron ($Fe^{2+}$) | ppm | 0.00 | 0.15 | 0.01 |
| pH | u | 8.64 | 8.85 | 8.86 |
| Phosphate | ppm | 1.56 | 13.13 | 0.43 |
| Sulfate | ppm | 456.33 | 102.01 | 749.95 |
| Chloride | ppm | 2800.00 | 4056.55 | 15555.96 |
| Nitrate | ppm | 8.02 | 9.92 | 77.15 |
| Phosphate | ppm | 58.37 | 36.02 | 348.73 |
| | ppm | 644.84 | 644.84 | 3539.00 |
| Barium | ppm | 0.01 | 0.01 | 0.06 |
| Potassium | ppm | 47.1 | 42.3 | 233.7 |
| Manganese | ppm | 0.05 | 0.02 | 0.1 |
| Strontium | ppm | 0.5 | 0.84 | 4.02 |
| Acetic acid | ppm | 58.06 | 0.00 | 0.00 |
| Propionic acid | ppm | 6.63 | 0.00 | 0.00 |

| | | | | |
|---|---|---|---|---|
| pH | - | 7.75 | 8.5 | 8.2-8.5 |
| Conductivity | mS.cm$^{-1}$ | 12.3 | 12-12.5 | 50-52 |

**Table 2S:** Electrolyte differences for the high salinity reactors between the time 0 (before the propionate spikes) and the time1 (after all the propionate spikes).

| Analyses | Mean Reactors A, C, E (Time 0) | Standard deviation | Mean Reactors A, C, E (time 1) | Standard deviation | t-test |
|---|---|---|---|---|---|
| Ammonium | 101.60 (ppm) | 19.36 | 133.48 | 13.63 | 0.05 |
| Conductivity | 54.25 (mS cm$^{-1}$) | 0.19 | 52.49 (mS cm$^{-1}$) | 3.75 | 0.49 |
| Iron ($Fe^{2+}$) | 0.02 (ppm) | 0.02 | 0.07 (ppm) | 0.07 | 0.47 |
| pH | 8.32 (U) | 0.01 | 8.59 (U) | 0.17 | 0.10 |
| Phosphate | 5.99 (ppm) | 2.64 | 10.51(ppm) | 1.27 | 0.14 |
| Sulfate | 740.07 (ppm) | 58.09 | 663.52 (ppm) | 57.10 | 0.08 |
| Chloride | 13301.53 (ppm) | 264.08 | 12077.84 (ppm) | 3002.79 | 0.63 |
| Nitrate | 67.49 (ppm) | 5.48 | 70.28 (ppm) | 19.55 | 0.78 |
| Phosphate | 152.86 (ppm) | 97.62 | 55.62 (ppm) | 39.85 | 0.23 |
| Sulfate | 2600.25 (ppm) | 275.78 | 1911.70 (ppm) | 885.77 | 0.21 |

| | | | | | |
|---|---|---|---|---|---|
| Barium | 0.07 (ppm) | 0.00 | 0.06 (ppm) | 0.01 | 0.18 |
| Potassium | 212.28 (ppm) | 7.88 | 212.22 (ppm | 19.27 | 1.00 |
| Manganese | 0.25 (ppm) | 0.02 | 0.19 (ppm) | 0.06 | 0.23 |
| Strontium | 3.32 (ppm) | 0.05 | 2.06 (ppm) | 1.80 | 0.35 |
| Acetic acid | 0.00 (ppm) | 0.00 | 0.00 (ppm) | 0 | NA |
| Propionic acid | 0.55 (ppm) | 0.90 | 0.00 (ppm) | 0.00 | 0.40 |
| pH | 7.07 (U) | 0.20 | 7.24 (U) | 0.99 | 0.78 |
| Conductivity | 43.29 (mS cm-1) | 2.18 | 52.62 (mS | 5.53 | 0.16 |

**Table 3S:** Electrolyte differences for the low salinity reactors between the initial time 0 (before the propionate spikes) and the time 1 (after all the propionate spikes).

| Analyses | Mean Reactors B, D, F (Time 0) | Standard deviation | Mean Reactors B, D, F (Time 1) | Standard deviation | t-test |
|---|---|---|---|---|---|
| Ammonium | 98.41 (ppm) | 14.00 | 136.26 (ppm) | 11.30 | 0.03 |
| Conductivity | 15.25 (mS $cm^{-1}$) | 0.09 | 13.32 (mS $cm^{-1}$) | 2.31 | 0.27 |
| Iron ($Fe^{2+}$) | 0.02 (ppm) | 0.01 | 1.00 (ppm) | 0.42 | 0.05 |
| pH | 8.16 (U) | 0.21 | 7.08 (U) | 0.24 | 0.00 |
| Phosphate | 8.31 (ppm) | 4.52 | 10.43 (ppm) | 1.51 | 0.534 |
| Sulfate | 134.03 (ppm) | 161.89 | 28.20 (ppm) | 5.11 | 0.36 |

| | | | | | |
|---|---|---|---|---|---|
| Chloride | 3676.40 (ppm) | 140.40 | 3181.21 (ppm) | 1206.00 | 0.59 |
| Nitrate | 9.54 (ppm) | 0.34 | 8.95 (ppm) | 3.88 | 0.83 |
| Phosphate | 13.72 (ppm) | 6.52 | 6.82 (ppm) | 2.49 | 0.24 |
| Sulfate | 47.18 (ppm) | 1.97 | 37.67 (ppm) | 14.13 | 0.71 |
| Barium | 0.00 (ppm) | 0.00 | 0.10 (ppm) | 0.02 | 0.01 |
| Potassium | 41.43 (ppm) | 0.96 | 34.70 (ppm) | 5.18 | 0.13 |
| Manganese | 0.16 (ppm) | 0.05 | 0.19 (ppm) | 0.05 | 0.25 |
| Strontium | 0.93 (ppm) | 0.01 | 0.70 (ppm) | 0.14 | 0.11 |
| Acetic acid | 2.09 (ppm) | 3.61 | 227.13 (ppm) | 82.38 | 0.04 |
| Propionic acid | 31.74 (ppm) | 27.51 | 121.96 (ppm) | 101.84 | 0.18 |
| pH | 6.71 | 0.17 | 6.18 (U) | 0.10 | 0.01 |
| Conductivity | 11.91 (mS $cm^{-1}$) | 0.43 | 16.62 (mS $cm^{-1}$) | 4.00 | 0.15 |
